# Versatile direct-writing of dopants in a solid state host through recoil implantation

**DOI:** 10.1038/s41467-020-18749-2

**Published:** 2020-10-07

**Authors:** Johannes E. Fröch, Alan Bahm, Mehran Kianinia, Zhao Mu, Vijay Bhatia, Sejeong Kim, Julie M. Cairney, Weibo Gao, Carlo Bradac, Igor Aharonovich, Milos Toth

**Affiliations:** 1 School of Mathematical and Physical Sciences, University of Technology Sydney, Ultimo, NSW 2007 Australia; 2 Thermo Fisher Scientific, Hillsboro, OR 97124 USA; 3 Division of Physics and Applied Physics, School of Physical and Mathematical Sciences, Nanyang Technological University, Singapore, 637371 Singapore; 4 Aerospace, Mechanical and Mechatronic Engineering, The University of Sydney, Sydney, NSW 2006 Australia; 5 Department of Physics & Astronomy, Trent University, 1600 West Bank Dr., Peterborough, ON K9J 0G2 Canada; 6 ARC Centre of Excellence for Transformative Meta-Optical Systems (TMOS), University of Technology Sydney, Ultimo, NSW 2007 Australia

**Keywords:** Nanoscience and technology, Optics and photonics

## Abstract

Modifying material properties at the nanoscale is crucially important for devices in nano-electronics, nanophotonics and quantum information. Optically active defects in wide band gap materials, for instance, are critical constituents for the realisation of quantum technologies. Here, we demonstrate the use of recoil implantation, a method exploiting momentum transfer from accelerated ions, for versatile and mask-free material doping. As a proof of concept, we direct-write arrays of optically active defects into diamond via momentum transfer from a Xe^+^ focused ion beam (FIB) to thin films of the group IV dopants pre-deposited onto a diamond surface. We further demonstrate the flexibility of the technique, by implanting rare earth ions into the core of a single mode fibre. We conclusively show that the presented technique yields ultra-shallow dopant profiles localised to the top few nanometres of the target surface, and use it to achieve sub-50 nm positional accuracy. The method is applicable to non-planar substrates with complex geometries, and it is suitable for applications such as electronic and magnetic doping of atomically-thin materials and engineering of near-surface states of semiconductor devices.

## Abstract (web summary)

Direct writing of active defects is a much sought-after step towards scalable quantum technologies. Here, the authors show a flexible approach by transferring momentum from an ion beam to thin films pre-deposited onto diamond surfaces (using films of Si, Ge, Sn or Pb) or fiber cores (using Eu).

## Introduction

Much of solid-state science revolves around modification of materials at the atomic scale, where site-selective control over the incorporation and formation of defects within a host lattice enables control of physical, chemical and optoelectronic properties. Ion implantation has become a key tool in this regard, allowing for the modification of material properties for nanoelectronics, spintronics and quantum photonics^1–4^. Of particular interest is the generation of optically addressable qubits such as colour centres in diamond, silicon carbide (SiC) or yttrium orthovanadate (YVO_4_) which are considered prime candidates for scalable quantum technologies^5–8^. Examples include the nitrogen vacancy (NV) centre^9^ and more recently the group IV elements^10–16^ in diamond that exhibit excellent optical and coherence properties suitable for quantum circuitry^17–19^.

Over the last few decades, significant efforts have been committed to the deterministic creation of colour centres in solid-state host materials by ion implantation^20–27^. While the technique is well established, there are fundamental constraints which limit its application in nanotechnology realisations. For instance, standard ion implanters employ beams that are too wide for direct-write patterning with a precision of tens of nanometres—requiring integration with mask-based lithographic techniques to achieve sub-micron lateral resolution. They are also generally ineffective for the implantation of foreign species into atomically thin materials such as graphene or transition metal di-chalcogenides^28^, especially when high positional accuracy and lateral resolution is needed. To overcome these intrinsic limitations, focused ion beam (FIB) systems which can operate at low energies and can perform direct writing have been developed. The main drawback of these systems, however, is the narrow range of atomic species available to them: most are limited to elements that can be implanted from a liquid metal ion source^29^.

Here, we demonstrate a feasible method that targets these limits. Stemming from recoil implantation and ion beam mixing^30–33^, the method utilises an inert primary FIB to implant secondary species into a solid host by transfer of momentum. When the method was proposed over half a century ago, ion beam mixing/recoil implantation was mostly studied with a focus on mixing at the interface of adjacent layers, without demonstration of working devices. Nevertheless, the technique combines features which are usually displayed—individually—by different approaches: it works for a large variety of elements, it is mask-free, and it is capable of incorporating dopants with high spatial resolution (approximately tens of nanometres) and at shallow depth (approximately few nanometres). By combining capabilities that typically require a range of instruments and methods, the demonstrated approach to recoil-implantation is complementary to existing ion implanters and FIB instruments, and will broaden their capabilities and reach. It works for a wide variety of chemical species, it is particularly appealing for multi-elemental implantation—both in the context of co-irradiations and serial irradiation—and it can be used on target samples with non-trivial spatial features and geometries. We showcase this versatility by fabricating a range of optically active, atom-like emitters from solid-state precursors in bulk diamond as well as at the apex of an optical fibre—the latter being a particularly challenging sample for standard mask-based lithographic techniques due its non-planar geometry.

## Results

### Concept

**Fig. 1:**
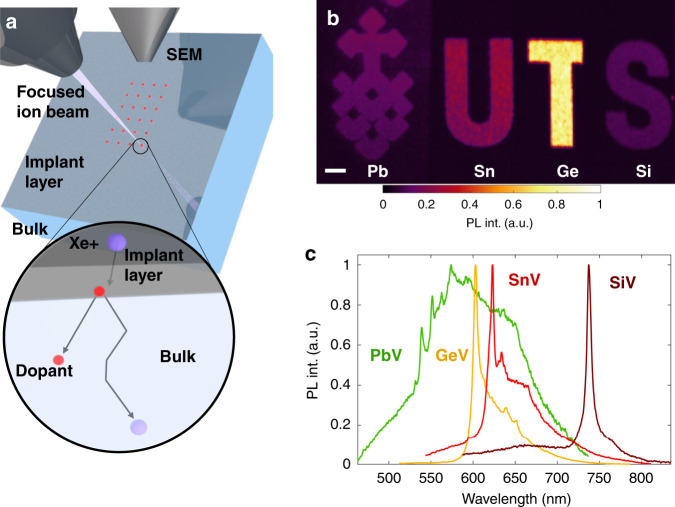
Creation of colour centres in a diamond target by recoil-implantation. **a** Schematic illustration of the process inside a standard electron-ion dual beam microscope. The inset shows an atomic picture of the process: momentum is transferred from inert primary ions to the atoms of a thin film comprised of the dopant material, resulting in implantation of the latter into the underlying target. **b** Photoluminescence map of a pattern fabricated in a single region of diamond using four dopant species that were implanted by momentum transfer from an electrostatically scanned Xe^+^ beam. The emblem is patterned in Pb, and the letters U, T, and S in Sn, Ge, and Si, respectively. The scale Bar corresponds to 5 μm. **c** Photoluminescence spectra recorded from the implants in **b**, showing characteristic emissions from defect complexes that contain the Pb (green), Ge (orange), Sn (red), and Si (maroon) dopants.

To implement the recoil implantation technique, we employ a readily available, unmodified dual beam system consisting of a coincident scanning electron microscope (SEM) and a FIB with a xenon (Xe) plasma ion source, as shown in Fig. 1a. The doping is performed by site-selective Xe^+^ beam irradiation of a target that contains the desired dopant species precursor in the form of a thin film. At the atomic scale, momentum transfer from Xe (purple circles in Fig. 1a inset) to the precursor atoms (red circles) causes the latter to be implanted into the underlying target. The process can yield ultra-shallow implant profiles, which is desirable for many applications.

As a benchmark for illustrating the versatility of this technique, we choose diamond, with a primary focus on a range of group IV elements in the periodic table: silicon (Si), germanium (Ge), tin (Sn), and lead (Pb). The reasons for the choice of host and dopants are as follows. First, diamond is available as an ultra-pure material with a wide bandgap and exceptional chemical inertness, making it an ideal platform for investigations of extrinsic dopants. Second, colour centres in diamond, particularly the group IV-related negatively charged vacancy complexes, are currently front runner systems for a plethora of quantum photonic and quantum sensing applications^10^. Hence, a robust method to create these centres is desirable. Finally, these defects in diamond are optically active and can be imaged at a single-photon level. This, in turn, allowed us to establish the efficacy of the proposed technique in the extreme case of advanced material systems which require engineering of individual, isolated atomic point defects.

To perform the recoil implantation, the aforementioned group IV elements were deposited onto diamond using a standard sputtering technique (15-nm thickness). Then, a 30 keV, focused Xe^+^ ion beam was used to pattern the *University of Technology Sydney* logo, and the acronym “UTS” into the thin film sections using an ion beam fluence of 2.5 × 10^13^ cm^2^. After irradiation, the thin film was stripped using chemical processes and the diamond was annealed to activate the emitters (i.e. vacancy complexes of Pb, Sn, Ge and Si). Further specifications are given in the Methods section and in the Supplementary Note 1. Figure 1b shows a photoluminescence (PL) map of the implanted area. The UTS emblem and the letters U, T and S are clearly visible. Importantly, the emblem and each of the letters consist of a different dopant—Pb, Sn, Ge and Si, respectively—implanted using the same inert Xe^+^ ion beam. We stress that the doping is achieved by momentum transfer from the Xe beam to the aforementioned elements in the thin films deposited atop the diamond. The pattern is formed by electrostatic scanning of the ion beam with no additional lithography steps. The process is performed using a dual FIB-SEM system. All imaging and spatial alignments are therefore performed using the electron beam which is coincident with the ion beam at the diamond surface and serves as a passive imaging tool free from undesired implantation. Figure 1c shows PL spectra from the respective areas, featuring characteristic emissions of the four colour centres with zero phonon lines (ZPLs) at ≅550 nm (PbV), ≅620 nm (SnV), ≅600 nm (GeV) and ≅740 m (SiV). Remarkably, the colour centres are all optically active and bright, despite the fact that the dopant profiles are ultra-shallow, as is discussed below.

### Fluence and spatial control

**Fig. 2:**
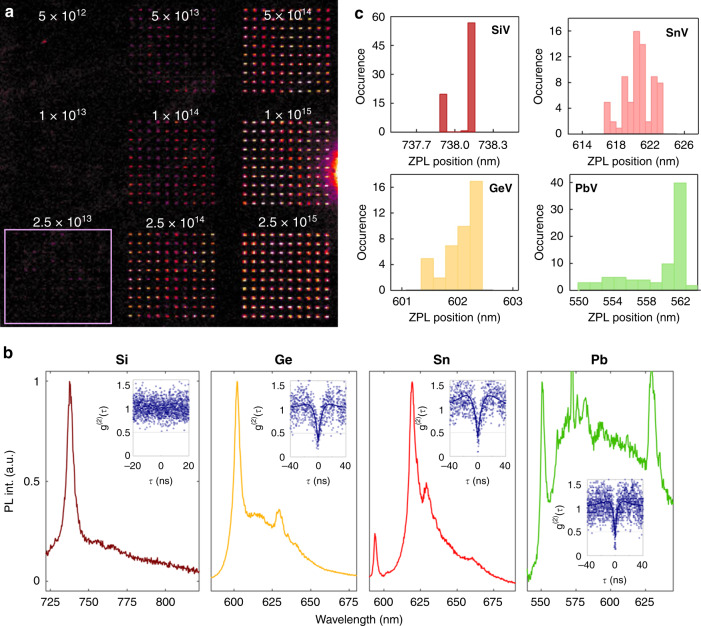
Spot arrays of dopants implanted into diamond as a function of Xe^+^ fluence. **a** Photoluminescence map of a representative array, here shown for Si dopants. The framed area shows the lowest fluence of Xe^+^ that yielded an unambiguous observable array in this case. The number above each array specifies the Xe^+^ fluence in units of cm^−2^. **b** Photoluminescence spectra of emitters from the lowest observable fluence of SiV (maroon), GeV (orange), SnV (red) and PbV-related emissions (green). An additional peak at 630 nm corresponds to the Raman signal of the utilised immersion oil. Insets show second order correlation measurements (background corrected), illustrating the quantum nature of the colour centres. **c** Histograms showing variability in ZPL wavelength in spot arrays of the colour centre ensembles.

We now turn to an in-depth analysis of the technique and the fabricated dopants, starting with the photophysical properties of the emitters. For this purpose, spot arrays (10 × 10) were patterned as a function of Xe^+^ fluence, which was varied logarithmically for each of the aforementioned dopants. Analysis of the implanted areas was then conducted at room temperature using a confocal setup with an oil-immersion objective. A representative map of a Xe^+^ fluence-dependent implantation series is shown in Fig. 2a. The image refers specifically to the case of silicon-vacancy (SiV) emitters, but identical arrays were also patterned for the other three elements (GeV, PbV and SnV). For all four elements, we observe the emergence of the corresponding colour centre emission beyond a particular ion beam fluence (specified in Fig. 2a). We note that the optical signals emerge after an annealing step, which promotes the formation of the fluorescent defect complexes, i.e. impurity atom adjacent to vacancy(ies), displaying PL emission. The observation of the emission above a particular Xe^+^ fluence is related to the stochastic nature of the activation process, further discussed in the Supplementary Note 2.

The ion beam fluence needed to form an unambiguous observable pattern, specific to the 15-nm dopant film thickness used in our experiments, is on the order of 2.5 × 10^13^ cm^−2^, 2.5 × 10^13^ cm^−2^, 5 × 10^13^ cm^−2^, and 5 × 10^13^ cm^−2^ for Si, Ge, Sn and Pb, respectively. This trend with atomic weight is intuitive given that the Xe^+^ ions mill the 15-nm films via sputtering and simultaneously implant the dopants into the underlying diamond via momentum transfer. Additional details about the creation yield and further characterisation (atomic force microscopy (AFM) and PL) are presented and discussed in the Supplementary Note 2 and 3, respectively. We note that co-implanted Xe ions may also form luminescent colour centres in diamond^34^, yet, their spectral features appear at 795 and 815 nm and do not overlap spectrally with the studied centres. Moreover, throughout the fluence range used in Fig. 2, the Xe-related emission was not observed in PL spectra. Further details are provided in the Supplementary Note 4.

Throughout the implanted arrays (fabricated as a function of Xe^+^ fluence), prominent ZPLs are observed at 738 nm (SiV), 602 nm (GeV), 620 nm (SnV) and 550 nm (PbV), as shown in Fig. 2b, which are characteristic of the respective colour centres. We note that an additional broad peak at 630 nm appearing in some of the spectra is the Raman signal of the immersion oil (Supplementary Note 5). Also, in the case of SnV, an additional peak at 595 nm is present, associated with intermediate defect configurations^35^. To confirm the quantum nature of the emitted light at the lowest implantation fluence, a second order autocorrelation measurment, (*g*^(2)^(τ)), was recorded from each of the emitters. The background-corrected *g*^(2)^ (τ) curves are shown as insets in Fig. 2b. A clear dip below 0.5 at zero-delay time is observed for GeV, SnV and PbV-related emitters, confirming the single-photon nature of the implanted colour centres. We did not observe anti-bunching from the fabricated SiV colour centres, most likely due to their very low quantum efficiency^36^.

The excited state lifetimes of the created defects are in accord with previously reported values—(1.5 ± 0.1) ns, (4.7 ± 0.1) ns, (3.9 ± 0.1) ns, (3.5 ± 0.2) ns, for SiV, GeV, SnV and PbV centres, respectively (Supplementary Note 6)^10^. Furthermore, resonant excitation at cryogenic temperatures was performed on some of the emitters. Narrowband linewidths on the order of 5 GHz were observed for the GeV and the SiV colour centres, which is promising for practical applications (Supplementary Note 7). For applications relying on the coherence of the spin state of these defects, further post-processing steps (e.g. high-pressure, high-temperature annealing) may be needed to remove residual crystal damage.

We further analysed the ZPL spectral properties of the ultra-shallow emitters. Interestingly, the distribution of ZPL wavelength increases with the atomic weight of the dopant species, under the exact same annealing conditions. For instance, the SiV ZPL is almost always at the same wavelength ≅738 m, while the PbV and SnV ZPL vary over several nm. Furthermore, additional spectral lines (tentatively attributed to intermediate defect states) appear to a greater extent and with higher frequency for heavier implants, as discussed in the Supplementary Note 8^14,35,37^. Whilst some of these characteristics can be attributed to an increase in strain associated with dopant size, these results warrant further investigation, in particular towards optimisation of the annealing process^35,38^. Nonetheless, the recoil implantation technique is robust and can produce colour centres on demand, as demonstrated here with the group IV family in diamond.

### Spatial control and depth profile

**Fig. 3:**
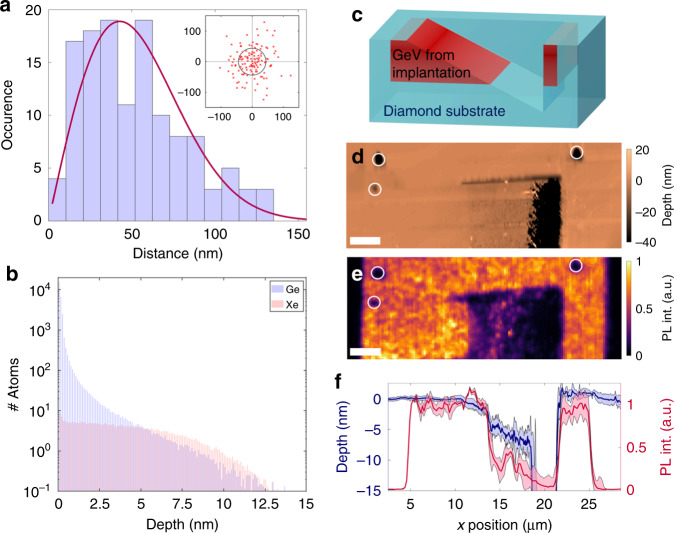
Spatial distribution of the dopants. **a** Distribution of the ensemble position (GeV) relative to the nominal implantation site, fitted by a Rayleigh function with a mode of (44 ± 4) nm. The inset shows directly the relative ensemble position. **b** Monte Carlo simulations of the depth distribution of Ge in diamond from a Xe^+^ recoil-implantation process and of the co-implanted Xe. **c** Schematic of the wedge edge to access the implantation profile of an area with the implanted species. **d** AFM map of the wedge prepared by EBIE. **e** PL map of the same Ge-implanted area. The positions of both maps were correlated by the three marked spots. Scale bars in **d**, **e** correspond to 2.5 μm. **f** Average profile of depth (blue) and PL (red) along the wedge feature. The shaded areas represent the standard deviation.

Next, we analyse the dopant depth profiles, as well as positional accuracy of the recoil implantation. Lateral precision was determined using PL maps of spot arrays of low fluence (5 × 10^13^ cm^−2^) GeV ensembles. The ensemble locations were determined by local fits of the PL signal at the nominal implantation sites to 2D gaussian functions and related to a fitted grid, as detailed in the Supplementary Note 9. The distance of the nominal position (i.e. the grid intersections) to the centre of the gaussian fit was calculated, yielding a Rayleigh distribution of the relative ensemble position, as shown in Fig. 3a, whereas the extracted relative position did not show any skewness in the *x* or *y* direction (inset). A fit to a Rayleigh distribution, yields a mode of (44 ± 4) nm, representative of the implant-placement accuracy of the technique. This value is better than that required for engineering of quantum emitters within photonic devices, e.g. in the high field region of a 2D photonic crystal cavity^21^.

To determine the ultimate lateral resolution of the technique, the above measure of accuracy should be considered in the context of the ion beam diameter, and that of straggle of atoms implanted. An experimental analysis of the ion beam is provided in the Supplementary Note 10. It should be noted that, in the case of the plasma FIB used here, the beam diameter is limited by the virtual source size and the ion beam focusing optics^39^. It can be improved in a number of ways, such as utilisation of a Gas Field Ionisation Source^40^, or a pierced cantilever^41^ with a hole that is smaller than the beam diameter and thus reduces it in the plane of the target. Beyond the beam diameter, a fundamental limit on resolution is imposed by the radial range and straggle of the implanted atoms. To illustrate the magnitude of this effect, we simulated the spatial distribution of Ge implanted into diamond from a 15-nm film by an ideal 30 keV Xe^+^ beam (i.e., a beam with a diameter of 0 nm). The simulation code and results, detailed in the Supplementary Notes 11 and 12, reveal that the radial range and straggle are ~6 and 4 nm, respectively—a minor component of the measured accuracy of (44 ± 4) nm.

The integration of FIB with a coincident high-resolution SEM in the same tool enables accurate and localised defect engineering at a particular target site (e.g. the high field region of a photonic resonator). We note that concurrent imaging using the SEM does not lead to any unwanted implantation or alteration of the diamond.

We now discuss the ultra-shallow implant depth profile. Monte Carlo modelling (described in the Supplementary Note 11) indicates that the technique yields dopants within the very top layer of the target surface, with a range and straggle (defined in the Supplementary Note 10) of ~0.2 and 0.6 nm, respectively. In more detail, the Ge depth distribution, shown on a log-linear scale in Fig. 3b, reveals that over 90% of the implanted species are located within a depth of 1 nm, and the tail extends to ~10 nm. This is in good agreement with the measured value of (8 ± 2) nm that is discussed below. For comparison, the depth distribution of the co-implanted Xe is also shown in Fig. 3b. The Xe depth profile is much more uniform, and not as concentrated as that of Ge within the top few nanometres of the diamond surface.

Experimentally, we cannot conclusively confirm by direct optical imaging that the emitters are ≤5 nm from the interface. Therefore, to verify the depths of the implanted emitters, we employed Electron Beam Induced Etching (EBIE) in an environmental SEM with H_2_O vapour as the etch precursor^42^. EBIE allows us to locally remove the top material of the implanted areas, without damaging the material underneath^43^. During EBIE the electron beam locally induces a chemical reaction with the environmental species (H_2_O) forming a volatile complex that either desorbs spontaneously or is removed by electron beam induced desorption. The rationale for the deployment of this technique is given by the fact that no further physical alteration of the implanted species is expected, as the etching process is driven chemically at the diamond surface.

Using EBIE, a wedge-shaped feature was etched along a square implanted area (3.1 × 10^12^ cm^−2^), as is shown schematically in Fig. 3c. In the following, we correlated the depth (AFM scan, shown in Fig. 3d) to the local fluorescence intensity (PL map, shown in Fig. 3e). Using three etched spots (white circles) as fiducial markers to correlate both maps, the average depth and PL profiles of the wedge were extracted (Fig. 3f). In particular, we observe a PL emission maximum from a mean depth of less than ≃1.5 nm in the section—along the *x*-axis—between the 11 and 13 μm mark. We note that the initial increase observable in the PL line profile may be caused by an increase in surface roughness. This hypothesis is supported by the observation that the increase in PL correlates with spot-like features clearly discernible in the PL map in Fig. 3e.

Consequently, as the mean depth drops below 1.5 nm, the PL intensity decreases sharply to less than 50%, in the section along the *x*-axis, *x* ≅ 13–14 μm and down to 20% at about (5 ± 2) nm at *x* ≅ 15 μm. However, a residual PL signal is still present at a depth of (8 ± 2) nm, which is in good agreement with the tail of the simulated depth profile (Fig. 3b). Specifically, at a depth of ≅8 nm, the number of Ge atoms drops below 1, which in combination with a conversion efficiency on the order of ≅1%^22^ makes it unlikely that colour centres are created beyond this depth. We note that the simulations do not account for channelling, which is likely insignificant, as indicated by an analysis of the simulated Ge atom momentum distribution at the film-diamond interface. Specifically, the momentum analysis shown in the Supplementary Note 12 reveals that the strong forward-directionality of the Xe^+^ beam is not retained by the Ge atoms.

Returning to Fig. 3f, the luminescence signal is restored at *x* ≅ 22 μm, corresponding to the end of the etched wedge. As one progresses further along the *x*-axis, the luminescence drops again to zero at the edge of the implanted area at *x* ≅ 25 μm.

### Implantation into restrictive geometries

**Fig. 4:**
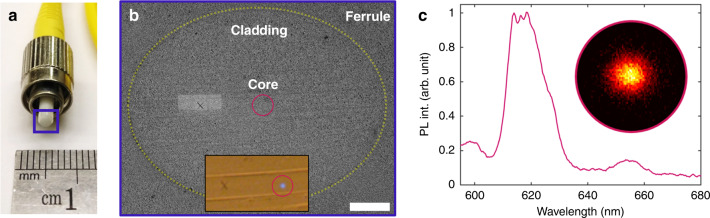
Implantation of rare earth ions into a commercial optical fibre. **a** Picture of the fibre used in this experiment. The end face is highlighted. **b** SEM image of the fibre end face during the implantation process. The yellow outline marks the boundary of the cladding to the ferrule, which was used to identify the core. The inset shows the core (back illuminated) relative to a correlation marker. The scale bar corresponds to 20 μm. **c** Emission of the implanted Eu^3+^, excited through the other fibre end (PL map shown as inset).

Next, we demonstrate another key feature of the recoil implantation technique by implanting a specific site of a target that has a restrictive geometry. Most common implantation methods do not use FIBs, but instead achieve site-specificity using mask-based lithographic techniques that are often inapplicable to relatively small, high aspect ratio, non-planar samples. To showcase how this constraint is overcome in an extreme example, we implanted a rare earth element, Europium, into the end face of a commercial single-mode optical fibre (Fig. 4a). We choose Europium to emphasise three key features. (1) Europium is heavier than Xe, which together with the Pb data in Figs. 1 and 2 shows that the technique is applicable to most elements in the periodic table. (2) The implantation of Eu at low energies is challenging by conventional methods since it is difficult to form the stable negative Eu ions needed by some ion implanters^44^. (3) The integration of rare earth elements with optical fibres is generally sought after for integrated photonics.

Analogous to the above experiments, a thin film of Europium was deposited on the fibre end. The fibre was then mounted in the FIB-SEM dual beam microscope and the fibre core was identified using the SEM, as shown in Fig. 4b. The inset of Fig. 4b is an optical image of the fibre, with the core lit up in blue by back-flowing light from a flashlight through the opposite end of the fibre. The mark “x” is a fiducial marker used to correlate the optical and SEM images. To implant the Eu, the core of the fibre was irradiated using a Xe^+^ fluence of 4.1 × 10^13^ cm^−2^. This value was determined beforehand by implantation into SiO_2_ (see Supplementary Note 13). Subsequently, the thin film was removed and the implanted area was investigated by PL analysis. As shown in Fig. 4c, we observed the emission related to the ^5^D_0_ → ^7^F_2_ transition of the Eu^3+^ ion centred at a wavelength of ≅620 m. The emission is confined to the core of the fibre, as shown by the confocal PL map obtained by exciting the Eu^3+^ from the back of the fibre.

## Discussion

To conclude, we demonstrated versatile creation of shallow implants in a solid-state host using recoil implantation. This is achieved by utilising momentum transfer from a Xe^+^ FIB to atoms of a thin target film deposited on top of a target of choice. In our work this is demonstrated by implantation from solid thin films deposited on the sample surface (diamond in our case). A compelling aspect of the technique is the ability to achieve ultra-shallow implants. We used the method to create optically active, atom-like defects in diamond and demonstrated their formation within the top 6 nm from the surface. To demonstrate the applicability of the technique to non-conventional samples, we engineered rare earth elements directly in the core of a single mode optical fibre. Beyond the field of quantum technologies, the recoil implantation technique is appealing for shallow doping of single electron transistors^45^, deterministic direct writing of near-surface dopants^46,47^ or controlled introduction of magnetic elements for magnetism at the nanoscale^48^. Finally, one of the biggest advantages of this technique is the ability to engineer selected defects in atomically thin materials, which by definition requires ultra-shallow implants.

## Methods

### Sample preparation

A CVD-grown electronic grade diamond (<1 ppb Nitrogen) was purchased from Element Six. Before all subsequent experiments were carried out, the sample was cleaned in hot (150 °C) Piranha Acid (H_2_SO_4_:H_2_O_2_ (30%) 2:1) for at least 2 h. All thin film depositions were carried out in a lab-built magnetron sputter deposition chamber, pumped to a pressure of 1 × 10^−5^ Torr or lower before deposition. For deposition, argon was introduced with a pressure of 1.5 × 10^−3^ Torr and a plasma was ignited, for which the power was adjusted to yield a deposition rate of 0.5 Å s^−1^. For the implantation experiments discussed in Figs. 2 and 3 the diamond was coated at four different corners with the respective thin films, whilst being masked during subsequent deposition steps to avoid cross deposition of different materials. For the patterning of the UTS logo, thin films were deposited on rectangular sections, lithographically defined using EBL as described in Supplementary Note 1. For implantation into an optical fibre or SiO_2_, a 15-nm Eu film was deposited on top in a thermal evaporator using Eu pellets. Sputter targets and evaporation material were purchased from Changsha Xinkang Advanced Materials Co., Ltd with a purity of 99.99% or higher.

### FIB irradiation

All ion beam irradiations were carried out in a Thermo Fisher Scientific Helios G4 PFIB with exchangeable plasma ion source. For irradiation, a Xe beam was used with an acceleration voltage of 30 kV and current of 10 pA. For irradiation of spot arrays and the UTS logo, custom patterning files (stream files) were created, defining the time per spot for individual spots (pixels). Box irradiations as discussed in the Supplementary Note 3 were performed, defining squares with 4-μm length and increasing the number of passes during the irradiation, using the built-in pattern generator.

### Post irradiation treatment

For experiments on diamond, after irradiation with the Xe^+^ FIB, the coated thin films were chemically stripped. We used subsequently two cycles of various solutions with intermittent water rinsing in between. First, the sample was held in KOH (30 min., 50 °C, 30 wt.%), then HCl (30 min., 75 °C, 37 wt.%), followed by Piranha Acid (30 min, 150 °C H_2_SO_4_:H_2_O_2_ (30%) 2:1) to efficiently eliminate any residue of thin film coating on the substrate surface. After the chemical treatments the sample was annealed in a tube furnace (Lindberg Blue Mini-mite) under high vacuum with pressure lower than 2 × 10^−6^ Torr for the entire annealing cycle. The temperature was ramped up to 950 °C and held for 2 h. Then the furnace was cooled down to room temperature before breaking vacuum. After annealing the sample was again cleaned in hot (150 °C) Piranha Acid (H_2_SO_4_:H_2_O_2_ (30%) 2:1) for at least 2 h and stored in a desiccator in between experiments. For experiments on SiO_2_ and the optical fibre, the Eu thin film was removed after implantation in warm HCl 50 °C and subsequently water. No annealing step was performed to activate the Eu^3+^ related emission.

### PL measurements

All PL measurements were performed on lab-built confocal setups. The UTS logo was characterised using a 405-nm, 1-mW cw laser as the excitation source, which was directed through an arrangement of a dichroic mirror, scanning mirror and lens relay system and focused on the sample surface by a 0.9 NA air objective. PL from the sample was collected from the same objective and directed into a multimode fibre guiding the signal either to an APD (excelitas) for mapping or to a spectrometer (Princeton Instruments). Lifetime measurements were conducted on the same configuration using either a 512 nm (for Si, Ge, Sn) or 405 nm (for Pb) pulsed laser diode as the excitation source. The fluorescence was correlated to the laser pulse using a correlator (Pico Harp 300, Picoquant). For the characterisation of the fibre core, the fibre was mounted in front of the air objective and a 2-mW, 532-nm cw laser was connected to the unimplanted end of the fibre to excite implanted Eu^3+^ in the core on the other end. At the same time, the collection was scanned in the same configuration. The characterisation of spot arrays and second order correlation measurements were done on a different setup. A 532-nm laser (cw, 2 mW) was used as the excitation source, and focused onto the sample by using an oil-immersion objective (1.3 NA). PL was collected through the same objective and coupled into a single mode fibre. The signal was either guided to a Spectrometer (Andor), or a 50/50 fibre splitter with the respective outputs connected to an APD. Their signals were then correlated by a time correlator (Swabian Instruments).

### Electron beam induced etching (EBIE)

EBIE of the diamond was done in a Zeiss EVO, under 15 keV, 2 nA and 100 mTorr H_2_O vapour. A rectangular area (16-μm length, 10-μm width) was irradiated (5-μs dwell time, 20-nm point pitch) in a raster scan. The length of the rectangle was reduced in subsequent steps by 2 μm, whilst the time/ area (≃1 s μm^−1^) for each rectangle was held constant, thus achieving a linear fluence gradient resulting in the shown etch profile. After etching the diamond was again cleaned in hot (150 °C) Piranha Acid (H_2_SO_4_:H_2_O_2_ (30%) 2:1).

### AFM measurement

AFM measurements were done on a Park XE-7 AFM. Post-processing of AFM data was done in XEI and Gwyddion^49^ to remove artefacts of the scan and to extract height profiles.

## Supplementary information

Supplementary Information

## Data Availability

The data that supports the findings of this study are available from the corresponding author upon reasonable request.
