# Supplementary material for: Versatile direct-writing of dopants in a solid state host through recoil implantation
Source: Nat Commun. 2020 Oct 7;11:5039. doi: 10.1038/s41467-020-18749-2 (PMC7541527; doi:10.1038/s41467-020-18749-2)
Supplement: Supplementary file 1 — Supplementary Information [file 41467_2020_18749_MOESM1_ESM.pdf]

Supplementary Information - Versatile direct-writing of dopants in a solid state host through recoil implantation

Fröch et al.

Supplementary Note 1 - Fabrication Schematic

A schematic of the generic fabrication steps is shown in Supplementary Figure 1. As described in the Methods section, CVD-grown electronic grade diamond (<1 ppb Nitrogen) was coated with thin films of Si, Ge, Sn and Pb (thickness = 15 nm) on its four respective corners. Then, areas were locally irradiated (spots and squares) with a Xe FIB at 30 kV and 10 pA and varying fluences, controlled by the dwell time. After FIB processing, thin films were stripped in KOH, HCl, and Piranha Acid. Subsequently the sample was annealed in High Vacuum and characterised by PL.

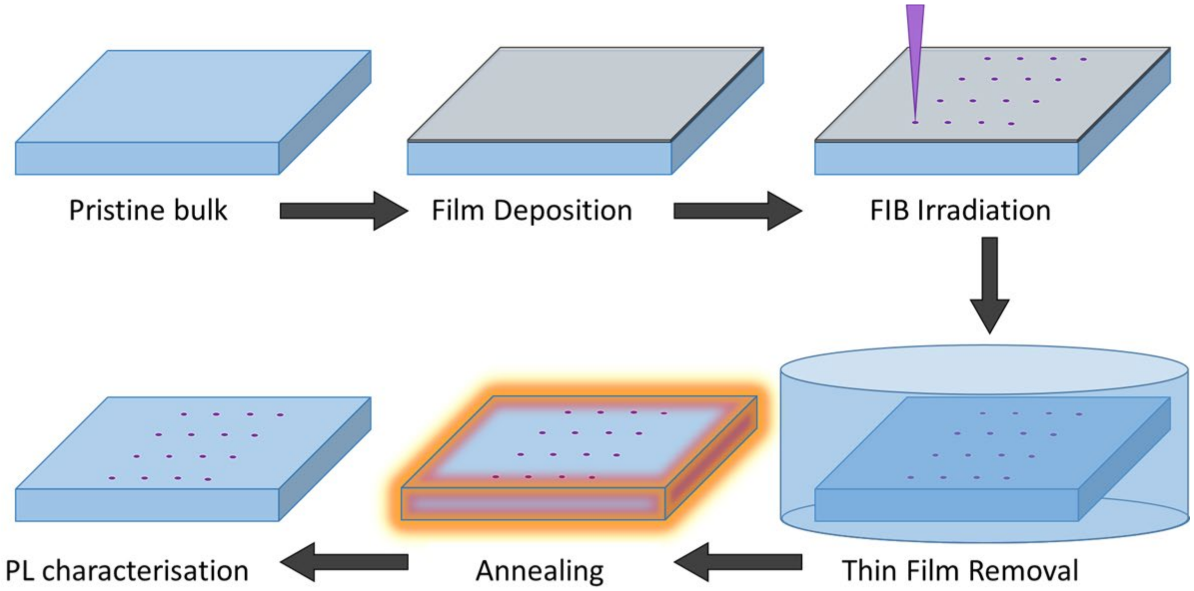

Supplementary Figure 1. *Schematic of the fabrication process for implantation utilizing momentum transfer. A pristine bulk sample is coated with thin films of the species to be implanted. A Xe ion beam is then used to locally irradiate the sample. The thin films are finally removed in KOH, HCL, and Piranha Acid. After thorough sample cleaning, annealing was used to activate colour centres, which were subsequently characterised in PL.*

For the creation of the UTS logo thin films were deposited in the following way. First the diamond sample was coated with a resist layer (PMMA), then a rectangular area was defined using EBL and subsequently a thin film was deposited. In the next step the resist was removed in warm acetone. Thus, only a small area of the entire sample would be covered with a thin film of a particular material. This cycle was then repeated, whilst a new area was placed next to the previously deposited one. This way, four neighbouring areas with thin films of Si, Ge, Sn, and Pb (in this order) were deposited. Each specific area was then identified in the Dual Beam Microscope using the SEM, shown in Supplementary Figure 2a. The different film types are apparent by SEM image contrast (the Ge thin film partially overlapped with the Si film, causing a thicker layer in this region). In a second patterning attempt (Supplementary Figure 2b), the top right part of the letter T was patterned over this area, and due to the thicker film implantation did not occur. Therefore, this letter appeared cropped on its top right part in the corresponding PL map (inset).

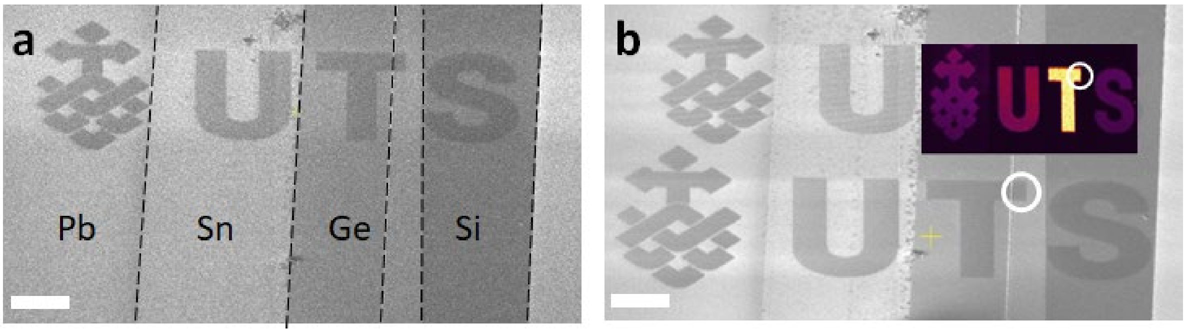

Supplementary Figure 2. *SEM images of an implanted area with different thin films. The same area before (a) and after (b) the patterning step that created the UTS logo that is presented in Figure 1b. The corresponding PL map is shown as an inset. The part which appeared cropped on the letter T is highlighted in the SEM image and PL map. Scale bars are 10 µm.*

Supplementary Note 2 - Creation yield

Here we provide a rough estimate of the average creation yield for the GeV, SnV and PbV colour centres deduced from the dependence of PL intensity on fluence. We extracted the pixel intensities at emitter sites from PL maps (such as the one shown in Figure 2). The average PL intensity as a function of the fluence for the various colour centres is shown in Supplementary Figure 3a. While these values for individual defects are comparable, we note that their absolute comparison should be taken with a high degree of caution, due to the utilisation of different spectral filters during the acquisition of the PL maps, used to maximise the contrast and clearly distinguish the spots. For each colour centre we observed the emergence of luminescence above a particular fluence, as mentioned in the main text, followed by a clear increase of intensity with fluence, which saturates for SiV and GeV at higher fluences, while no such saturation is observed in the case of SnV and PbV. The saturation is expected, as quenching is likely to occur at higher fluences due to increased damage. The emergence of emission above a certain fluence is also expected, due the stochastic nature of the colour centre activation process. For implantation from a FIB the conversion yield was reported to be ~4% for SiV[1], and ~0.4–0.7% for GeV,[2] respectively. However, in this case a focused ion beam was used, for which the conversion yield can be directly determined from the number of colour centres and the number of implanted ions. Since, in our case, the number of recoil implanted atoms does not directly correlate 1:1 to the primary Xe^+ ion fluence, we used the creation yield (colour centres generated per primary Xe^+ ion) as a comparable measure. In detail, the pixel intensity was related to the intensity of single emitters and a rough estimate of the created centres per Xe^+ ion was determined as shown in Supplementary Figure 3b. The number of created centres for SiV is not shown here, as it was not possible to unambiguously identify a single emitter experimentally. Therefore, the PL intensity of an actual SiV single emitter is likely lower, hence for similar average intensities, a higher number of colour centres as compared to other elements is expected. Comparing the different yields with each other, the lighter element Ge has a at least 2 times higher number of created centres per Xe ion than the other elements. Moreover, for all colour centres we observe the highest creation yield at a fluence of 5×10^{13}, followed by a reduction in the creation yield. This indicates that at higher fluences, the damage in the diamond lattice—introduced by primary Xe ions and recoil implanted atoms—was likely too high, causing quenching of the created colour centres. However, at this point it should be noted that all colour centres were created on the same diamond, which has undergone the same annealing

treatment. This annealing treatment (limited by our tube furnace setup), likely resulted in a higher conversion efficiency for lighter implanted atoms into an optically active defect, which may explain the higher creation yields for lighter elements.

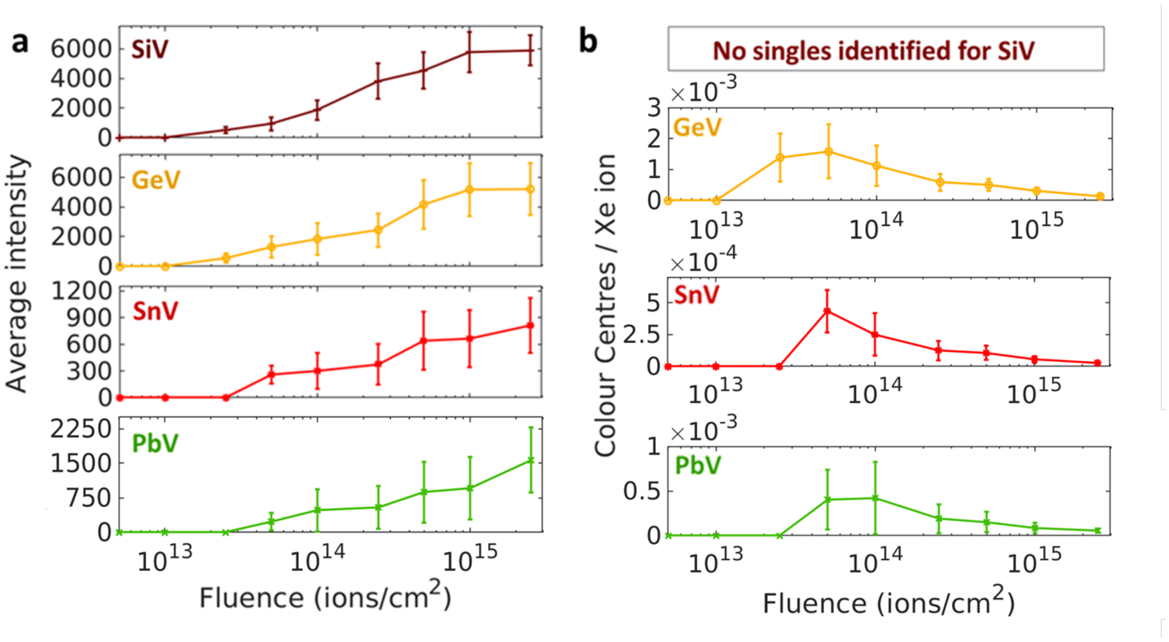

Supplementary Figure 3. *Determination of the creation yield for different colour centres versus* Xe^{+} *fluence. a) The average pixel intensity was extracted from PL maps. Error bars indicate standard deviation of the average. b) Colour centres created per primary Xe ion, estimated by comparison of the average intensity to the intensity of identified single photon emitters. Error bars indicate standard deviation of the average pixel intensity.*

Supplementary Note 3 - Further characterisation

To estimate the damage to the bulk diamond due to the implantation, we irradiated square areas (4 x 4) μm^2 with increasing fluences as shown in Supplementary Figure 4a. The primary Xe ion fluence of the first field was 1.6×10^{12} cm^{-2}, for each following field the fluence was doubled, with values of 3.1×10^{12} cm^{-2}, 6.2×10^{12} cm^{-2}, 1.3×10^{13} cm^{-2}, 2.5×10^{13} cm^{-2}, 5×10^{13}, 1×10^{14}, 2×10^{14}, 4×10^{14}, 8×10^{14}, 1.6×10^{15}, 3.2×10^{15} for fields 2–12, respectively. From the PL maps we observed, as the fluence increased, the PL intensity increased accordingly from fluence 1–4, followed by a decrease from 4–9 until the PL intensity increased again from 9–12. We note that this trend was observed for all elements. The initial increase can be attributed to a direct increase of the implanted species, leading to an increased concentration of luminescent colour centres. With further increase in fluence, we infer that degradation of the material leads to quenching of the PL signal. Note also that the thin film was exhausted during irradiation (removed by concurrent sputtering). The final increase in PL intensity is then related to damage-related luminescence.

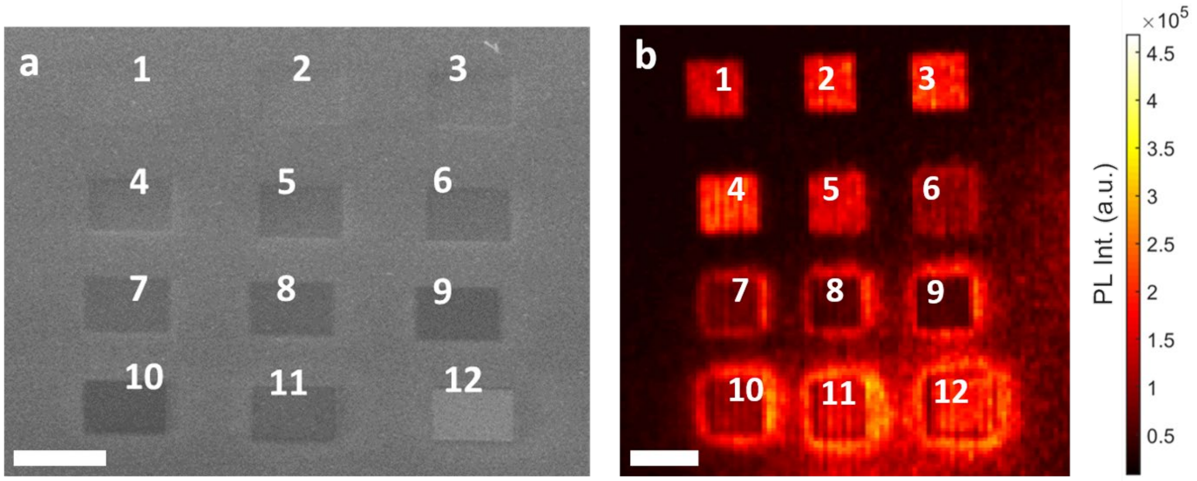

Supplementary Figure 4. *Square fluence series used to estimate the damage related to the technique. a) SEM image of a square series directly after irradiation (52° tilt). b) Representative PL map (Sn) of the fluence dependent series. Scale bars are 5 µm.*

For the lower fluence range, despite the relatively high number of primary ions required for emitter creation, we observed that pronounced damage is virtually absent in PL spectra (Supplementary Figure 5) or AFM profiles (Supplementary Figure 6) for Si (a), Ge (b), Sn (c), and Pb (d), respectively. From the PL spectra we observe that for low fluence (spectra 1) the background is lower in comparison to higher fluences, as seen particularly well in the case of Si, where essentially no PL is observed in the range 600 nm–700 nm. On the other hand, the diamond Raman line (1332 cm^{-1} / 572 nm and 2nd order 2664 cm^{-1} / 620 nm) is clearly present for all fluences and species. We also observe the emergence and increase of the G-band (1580 cm^{-1} / 581 nm) with increasing fluence. Whereas the former is indicative of the presence of diamond, the latter is direct evidence of damage in the form of sp^2- amorphisation. We note that even for high fluences (where the amount of sputtered material is significant), the diamond Raman line is present, as the implanted/ damaged layer is only present within a small section at the surface. At the highest fluence, clear formation of NV centres is observed (evidenced by the shapes of the PL spectra, resembling a ZPL at 637 nm with a broad phonon side band). We assume that despite their low natural concentration in the material, enough vacancies are formed ion beam to activate even the low concentration of nitrogen.

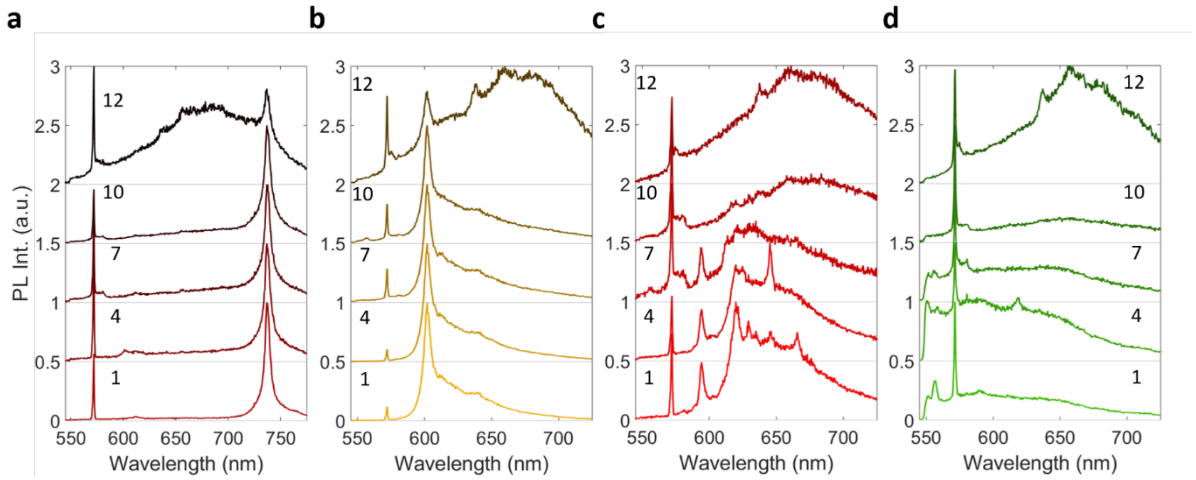

Supplementary Figure 5. *Spectra taken from the centres of the squares shown in* Supplementary *Figure 4. The number indicates the fluence mentioned in the text. (a) Si, (b) Ge, (c) Sn and (d) Pb.*

AFM scans of the implanted areas are shown in Supplementary Figure 6. Regions 1–4 are non-discernible from the surrounding region, evident by a non-changing height profile in the AFM map. With further increasing of the fluence, in regions 5–8 the height profile distinctly increases above the height level of the surrounding area. This increase is evidence of swelling, due to implantation from primary Xe ions, as well as from the target layer. Moreover, we observe that the amount of swelling appears to increase with the mass of the implanted species, i.e. for Pb the highest swelling can be seen for fluence 10 with a change in height of ≅15 nm, whereas for lighter elements this value is lower, ≅ 5 nm for Sn (fluence 9), ≅3 nm for Ge (fluence 8), and ≅1 nm for Si (fluence 7). Note that the depth and PL cannot directly be correlated to the implantation profile as discussed in Figure 3. As we demonstrated throughout this work, atoms can be implanted directly from a thin film into the underlying bulk, and already-implanted species may be buried deeper by subsequent momentum transfer. Therefore, it was crucial to utilize Electron Beam Induced Etching (rather than FIB milling), to avoid any subsequent alteration of the implantation profile.

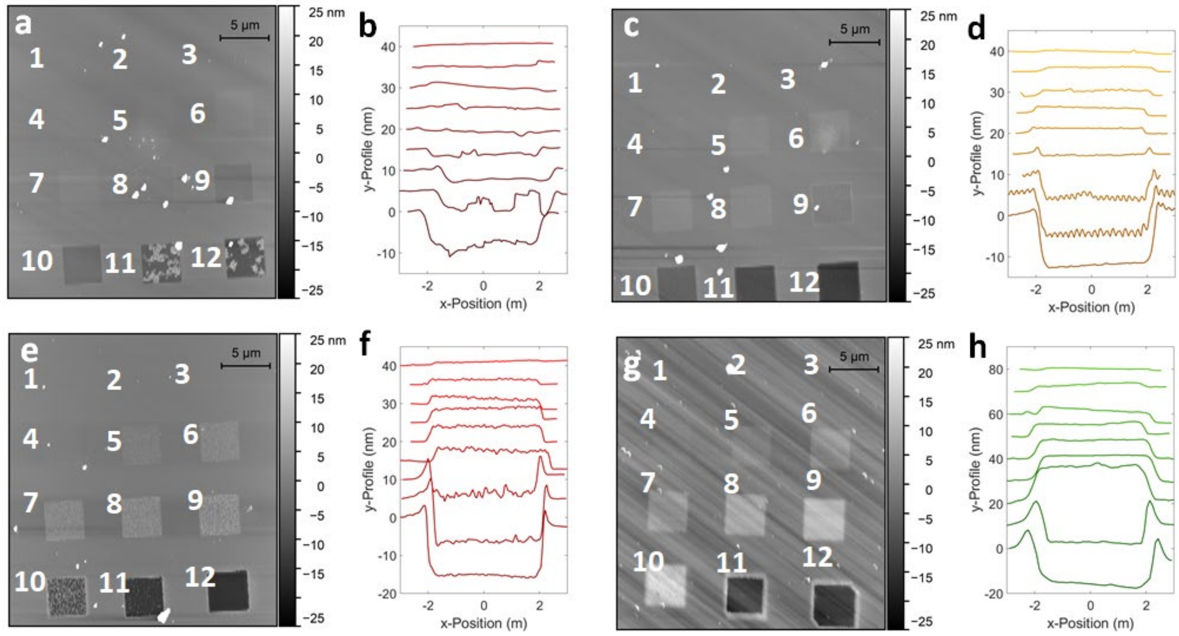

Supplementary Figure 6. *AFM scans and depth profiles of the implanted squares for Si (a) and (b), Ge (c) and (d), Sn (e) and (f), Pb (g) and (h). The numbers denote the aforementioned fluences. The depth profiles are offset in the y-direction for better visibility. For each graph, the first profile corresponds to fluence 4, the second to fluence 5 and so on. The diagonal streaks visible in the AFM maps are polishing marks of the as-purchased diamond.*

Supplementary Note 4 - Luminescence from Xe-implanted areas

As mentioned in the main text, Xe may also form a colour centre in diamond with ZPLs at 794 nm and 811 nm. However, we stress that during our measurements this emission was barely observable. Particularly, it does not overlap with any of the investigated emitters and furthermore its intensity is low in comparison to even the dimmest colour centre, the SiV, as shown in Supplementary Figure 7. Measurements were taken (using a 633 nm excitation laser) from areas with and without a thin film of Si at a fluence of 1.2×10^{13} cm^{-2} and normalised to the diamond Raman line at 696 nm. Notably the

Xe-related emission is not resolvable in the spectrum taken from the Si-implanted region, and even in the spectrum without thin film, its relative intensity is low compared to the SiV intensity.

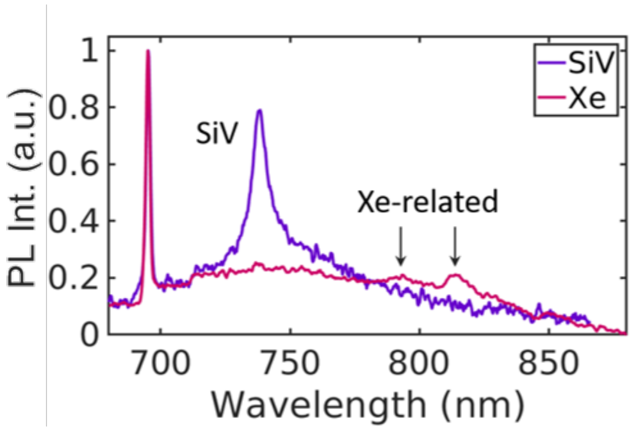

Supplementary Figure 7. *Comparison of the emission of the Xe-related colour centre to the SiV under 633 nm excitation.*

Supplementary Note 5 - Raman Oil Background

For the characterisation of the implantation spots, presented in Figure 2, we used a setup with an oil immersion objective to increase the collected signal. As described in the main text, the Raman signal of the immersion oil (Olympus Type F) was observable at ~630 nm. A spectrum, Supplementary Figure 8, for which the laser (532 nm) was focused above the sample directly shows the related PL signature. We note that this signal appears relatively strong for the recorded spectra, because the laser was focused at the oil-diamond interface, due to the shallowness of the implanted species.

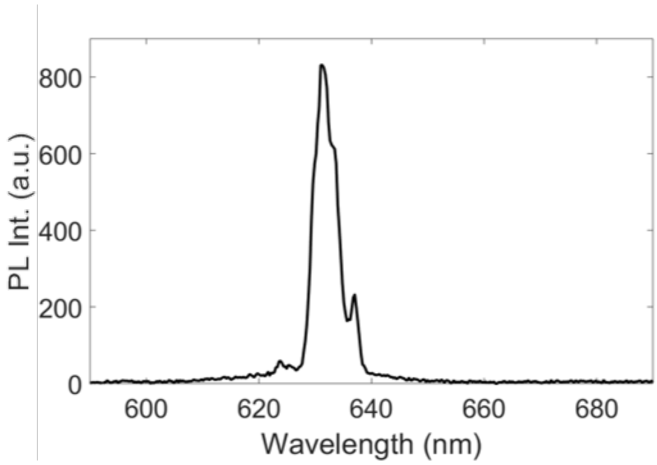

Supplementary Figure 8. *PL spectrum of the Raman signal related to the utilised immersion oil (532 nm excitation).*

Supplementary Note 6 - Ensemble Lifetimes

Time-resolved PL measurements were acquired using a 512-nm pulsed laser as excitation source and set to 20 MHz repetition rate. For acquisition of Pb-related colour centres, a 405 nm laser was used as the excitation source. For all ensembles, the PL was spectrally filtered, such that a spectral window of ~20 nm with ZPL at the centre was collected. The signal was acquired by an avalanche photo detector

and correlated to the laser using a high-resolution time correlator (Picoharp 300, PicoQuant). The decay curves are presented in Supplementary Figure 9 for Si (a), Ge (b), Sn (c), and Pb (d). The data was fitted with a single exponential decay function shown as a solid line in each figure. We obtain values for the lifetimes of (1.5 ± 0.1) ns for SiV, (4.7 ± 0.1) ns for GeV, (3.9 ± 0.1) ns for SnV, and (3.5 ± 0.2) ns for PbV. The presented measurements were done on ensembles of emitters, created with a fluence of 3.1×10^{12} cm^{-2}.

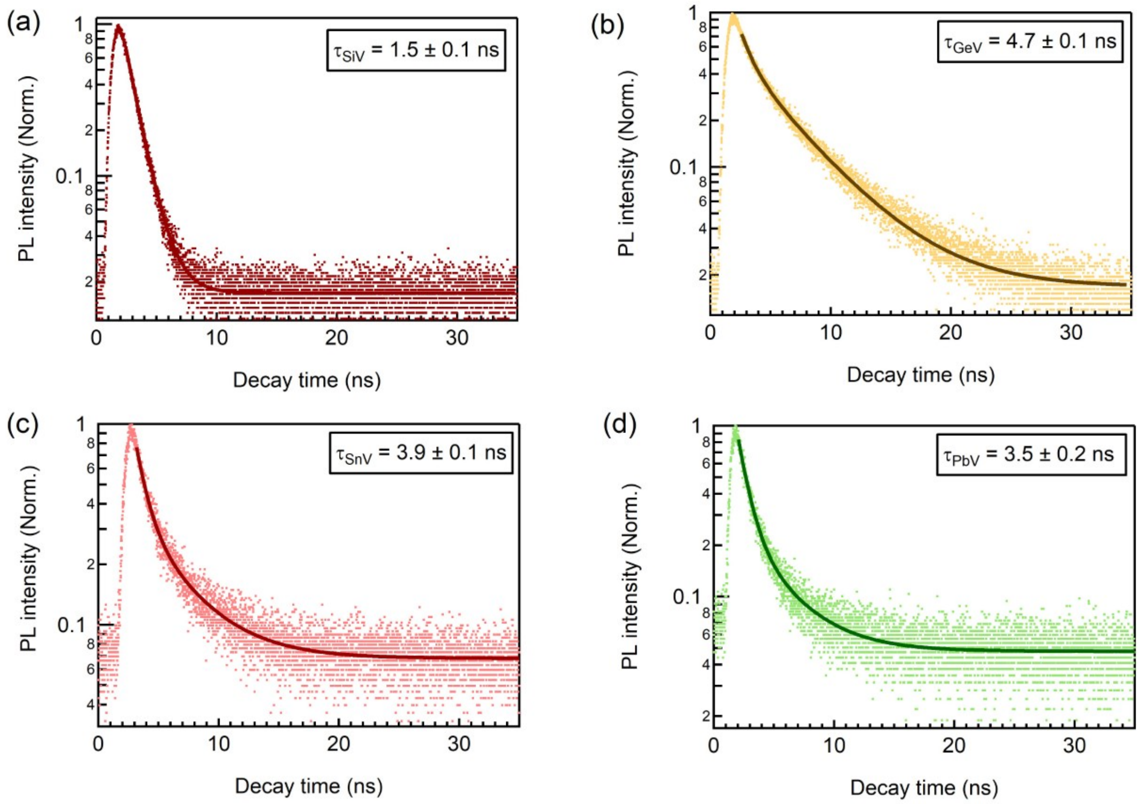

Supplementary Figure 9. *Lifetime measurements of implanted regions for SiV (a), GeV (b), SnV (c), and PbV (d) with the respective lifetimes determined from a single exponential fit, given in the top right corner.*

Supplementary Note 7 - Cryogenic Characterisation and resonant excitation

To gain further insights into the emitter characteristics, the spot-implanted sites were studied at a cryogenic temperature of 4 K. PL spectra as well as photoluminescent excitation (PLE) spectra, collected under excitation with narrowband lasers are shown in Supplementary Figure 10 for SiV in (a) and (b), GeV in (c) and (d), PbV in (e) and (f), respectively. The central laser frequency was set to 497.743 THz for GeV and 542.947 THz for PbV, respectively. From Lorentzian fits we obtained linewidths in PLE for SiV and GeV of 3.84 GHz and 4.73 GHz for the C Transition, respectively. Whilst for PbV spectral diffusion was observed and no conclusive value for the linewidth could be derived. This is likely related to the larger ion size of Pb, and the associated large strain in the diamond lattice, which may in the future be alleviated by better annealing treatments. Nonetheless, resonant excitation of the emitters can be clearly observed for the SiV and GeV with sub 5 GHz linewidths.

To the best of our knowledge, this is the first evidence of resonant excitation of sub-surface emitters. So far, resonant excitation was only carried out in bulk or in photonic nanocavities that still have a

thickness of over 100 nm. Hence, it is remarkable that resonant excitation is even possible, despite the proximity to the surface.

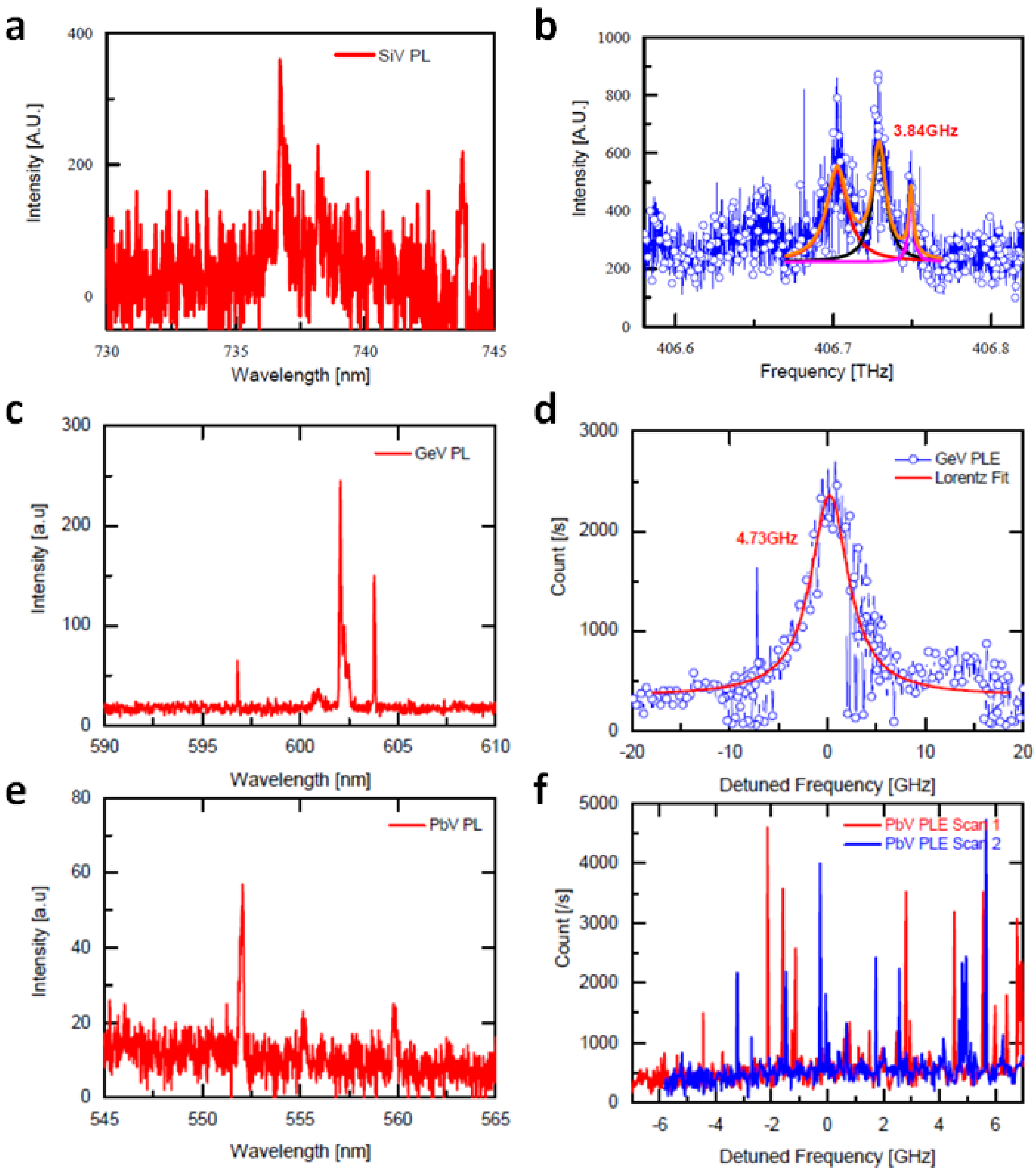

Supplementary Figure 10. *Characterisation at 4 K. PL (a) and PLE (b) spectra for spot implanted sites of SiV, (c) and (d) for GeV, (e) and (f) for PbV, respectively.*

Supplementary Note 8 - Observation of additional spectral lines

Besides the main ZPL, we observed additional lines at some distinct wavelengths. In Supplementary Figure 11 are the averaged spectra for different dosages (increasing bottom to top). The frequency of additional spectral lines increases with the size of the implanted species, which potentially indicates that these lines may be associated with intermediate defect states caused by additional strain in the diamond crystal. In particular, for SiV, as shown in (a), we only observed the main ZPL at 738 nm. For GeV, shown in (b), we observed primarily the main ZPL, and additional lines at ≅608 nm, ≅612 nm,

≅615 nm and ≅640 nm. Their occurrence was relatively low compared to the main ZPL, therefore these appear weaker in the spectra.

On the other hand, For SnV, the number of additional lines and their relative intensity increased dramatically. We almost always observe a peculiar line at ≅595 nm. Additionally, lines at ≅630 nm seem to appear, which are however superimposed on the aforementioned immersion oil Raman signal. Also, lines in the range 642 nm–647 nm, as well as ≅655 nm were observed. For PbV, the main ZPL was observed at 550 nm–560 nm, whilst further line-like features occur at ≅569 nm, ≅575 nm, ≅581 nm, as well as at ≅738 nm. Further spectral and correlative analysis is required to clearly identify the underlying nature of the mentioned lines. Recent advances to eliminate these lines in HPHT and subsequent overgrowth seem promising.[3, 4] In particular, the latter could potentially evolve into a powerful technique in this context, as it would allow to have a dopant layer localised with a depth variation of ≅ 6 nm.

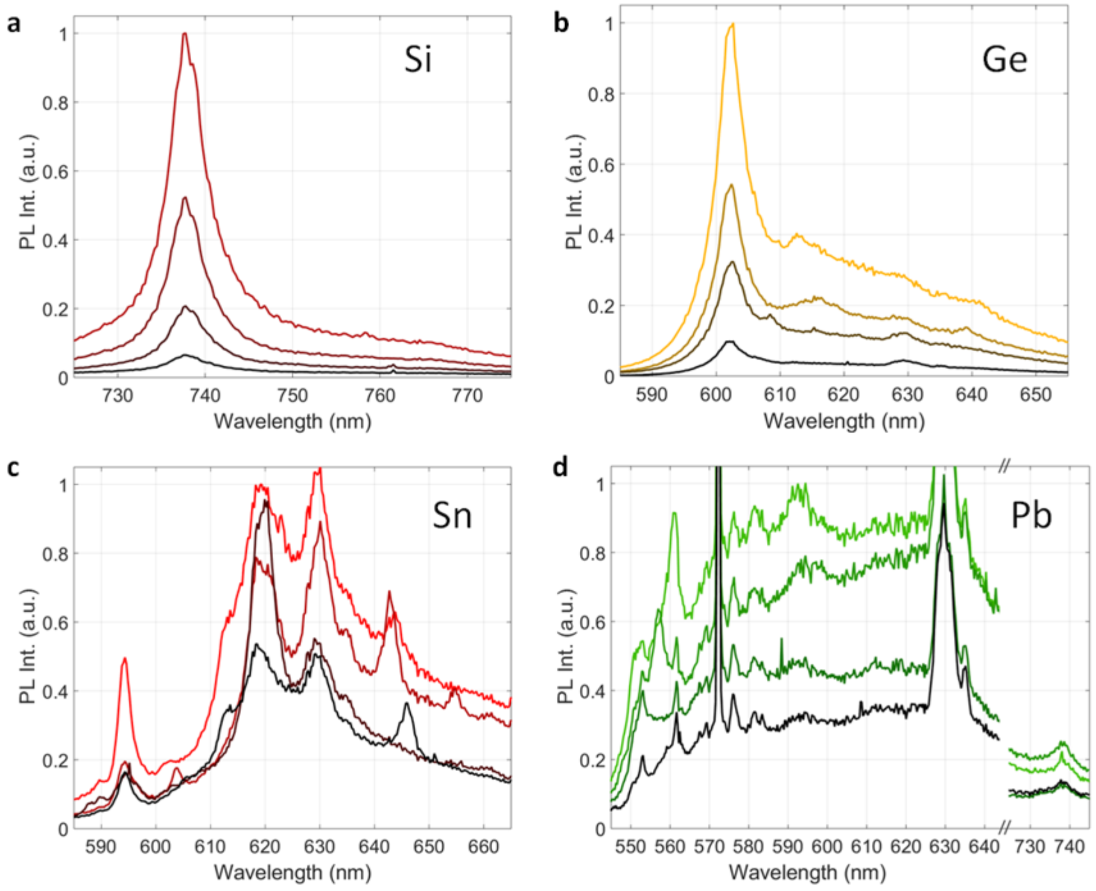

Supplementary Figure 11. *Averaged spectra over different dosages, normalised to the maximum of the highest fluence for Si (a), Ge (b), Sn (c), and Pb (d). The black lines correspond to the spectral average for the lowest observed fluence of the spot implantation series. The second lowest line (one shade brighter) corresponds to the next spot series and so forth.*

Supplementary Note 9 - Determination of Accuracy

To determine the precision of the created colour centres, we acquired a large area scan of a 10 x 10 spot array. To minimise the influence of setup-related aberrations (e.g. due to large map areas), as well as distortions due to drift (during the PL mapping), several subarrays of 4 x 4 points were considered. Horizontal and vertical lines were fitted through these points, with the constraint of

equivalent slopes and equidistant line offsets for individual subarrays. Gridline intersections were then taken as the nominal implantation sites. Examples of the subarrays of 4 x 4 spots (fitted by a 2D Gaussian) are shown in Supplementary Figure 12. Some spots were counted twice, due to their presence in different subarrays, therefore yielding a total number higher than 100 in Figure 3a. We note that the 4 top right and 4 bottom right points were excluded from the analysis, as we observed a systematic deviation compared to other sub grids, likely due to a too large aberration and drift at the corner of the PL map. Therefore, the sub grid in Supplementary Figure 12c, only considered rows 6–9.

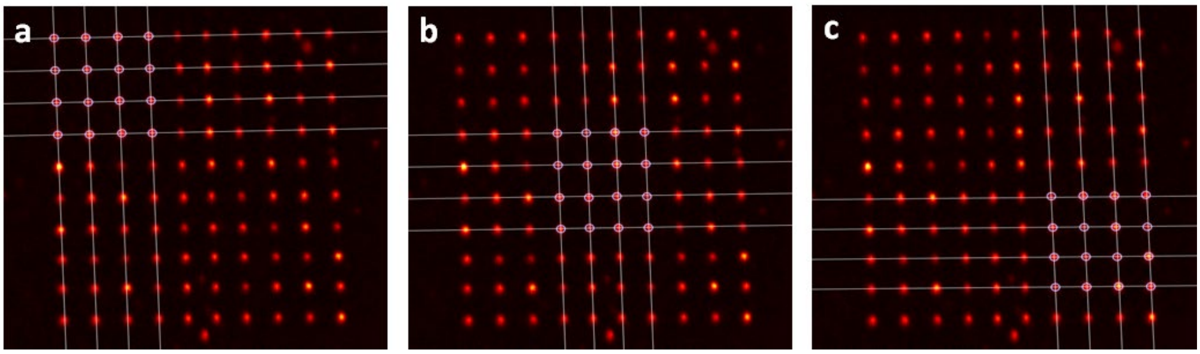

Supplementary Figure 12. *A large area scan (10 x 10 array), was analysed by fitting 8 sub grids (4 x 4). In (a), (b), and (c) three examples of subgrids are shown. We note that the subgrids in the most right section (right 4 columns) excluded the top and bottom 4 points, due to a systematic deviation.*

Supplementary Note 10 - Beam diameter, radial range and straggle

A decisive parameter for the precision of implanted species is the diameter of the focused ion beam. To determine this parameter for our specific equipment under working conditions, we irradiated spots on a plain silicon substrate, as shown in Supplementary Figure 13a. The spot dwell time was increased column-wise from left to right, to identify the lowest dwell time that would yield identifiable distinctive spots (red outline). These spots were then averaged (Supplementary Figure 13b) and fitted with a gaussian function (Supplementary Figure 13c). A comparison of both is displayed in Supplementary Figure 13d, showing the suitability of this function to reproduce the shape of the beam. The fit yielded a standard deviation of σ = 36 nm, giving a FWHM of 85 nm as a rough estimate of the beam diameter. We emphasise that the comparison of spots from the lowest observable dwell time is valid as, the spot irradiations as discussed in Figure 2 were done using alower dwell time (<2.5 ms) than the spots shown here (12.5 ms). The range and straggle (defined below) in depth and radial direction for the implanted Ge atoms were determined using the ISM code detailed in Supplementary Note 11, with a delta spike ion beam. We obtained the following values, depth range R_p = 0.12 nm, depth straggle σ = 0.19 nm, radial range R_r= 6.04 nm, radial straggle: σ_R = 3.64 nm.

We note that range and straggle are sometimes defined inconsistently in the literature. We used the following definitions:[5, 6]

Depth range $R_p = \sum_i ({x_i}/{N})$

Depth straggle $\sigma = [\sum_i ({x_i^2}/{N}) - R_P^2)]^{1/2} = \langle(\Delta x_i)^2\rangle^{1/2}$

Radial range $R_r = \sum_i {(y_i^2 + z_i^2)^{1/2}}/{N}$

Radial straggle $\sigma_r = \left[\sum_i \left(y_i^2 + z_i^2\right) / N - R_r^2\right]^{1/2} = \langle(\Delta r_i)^2\rangle^{1/2}$

where *x* is defined as the direction of the primary ion (Xe) and *y*, *z* are perpendicular to it and lie in the plane of the sample surface.

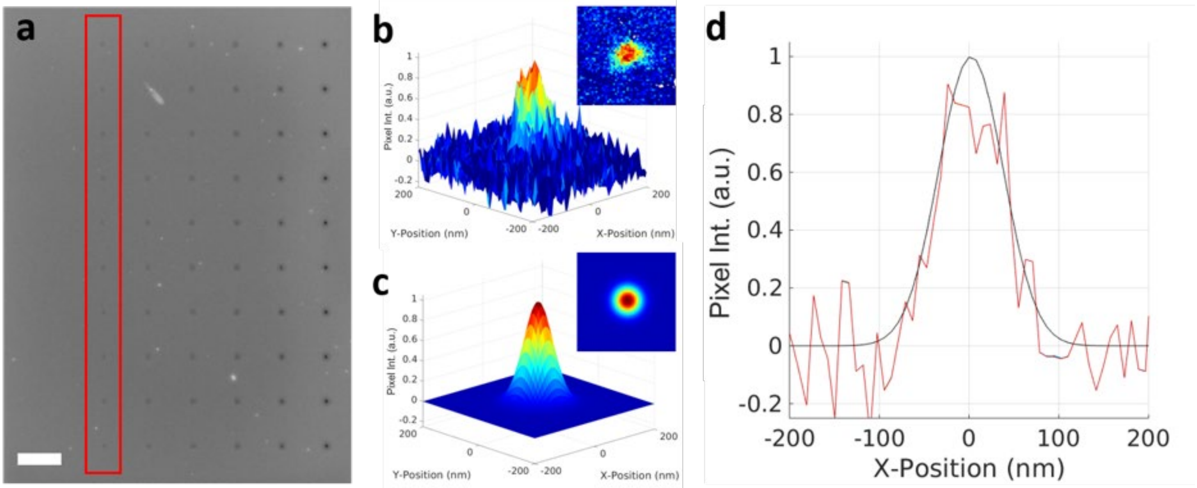

Supplementary Figure 13. *Determination of the beam diameter. a) A plain Si substrate was irradiated by spots with dwell times increasing column-wise from left to right. The scale bar corresponds to 1 µm. b) The normalised inverse pixel intensity of the average for the spots highlighted in (a). c) A Gaussian fit was used to approximate the ion beam shape. d) Direct comparison of measured and fitted beam shape.*

Supplementary Note 11 - Description of the ion-solid modelling code

The Monte Carlo ion-solid modelling (ISM) code used here is a multithreaded implementation of the binary collision algorithm (BCA), allowing fast computation as well as flexible definitions of surfaces and materials.[7] ISM has been validated against the commonly used SRIM program (Stopping and Range of Ions in Matter),[5] as shown in Supplementary Figure 14 for simulations of the implantation depth and straggle for Xe^+ ions at varying primary ion energy into graphitic carbon, further detailed in reference 7.

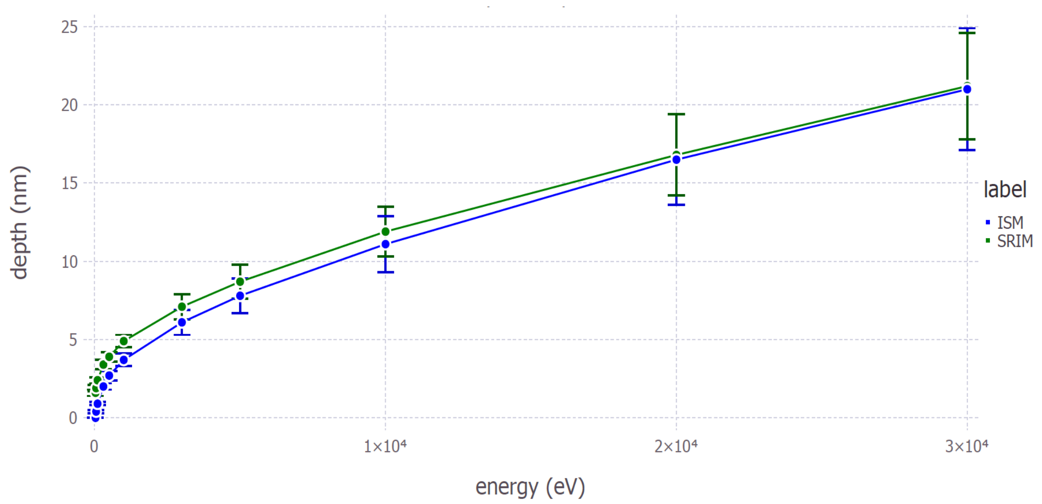

Supplementary Figure 14. *Comparison of range and straggle of the utilised ISM code with SRIM in dependence of the primary energy for Xe^{+} ions. The error bars correspond to the straggle of the implanted ions.*

Supplementary Note 12 - Angular distribution of recoil-implanted Ge

From the simulation of recoil doping of diamond from a 15-nm (amorphous) Ge film, we additionally extracted the angular distribution of the Ge atoms at the film-diamond interface. The distribution is shown in Supplementary Figure 15. The abscissa represents the angle relative to the normal vector of the diamond surface (described in the inset). Values from 0° to 90° correspond to Ge atoms that left the diamond and were scattered back into the Ge thin film, whilst entry angles from 90° to 180° correspond to atoms entering the diamond. The distribution peaks at ~140°, it is broad, asymmetric and lacks the forward-directionality of the incident Xe^{+} beam.

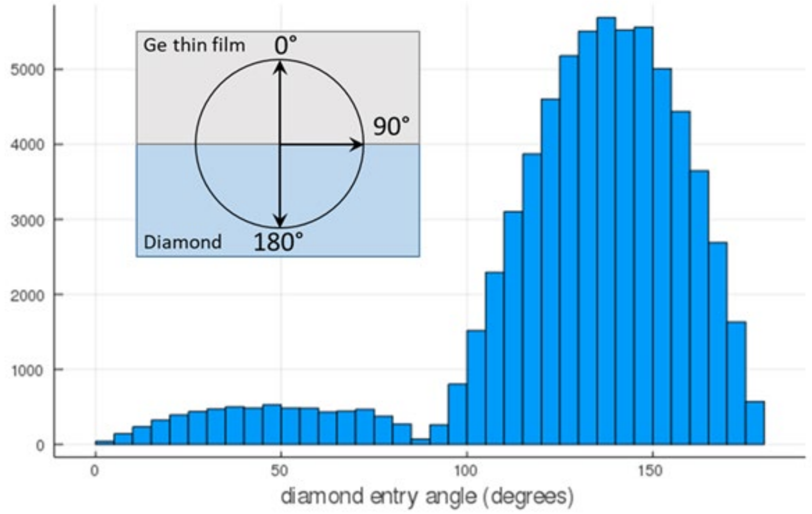

Supplementary Figure 15. *Angular distribution of Ge atoms trajectories as they cross the thin-film diamond interface. The orientation of the angle relative to the thin-film diamond interface is shown in the inset.*

Supplementary Note 13 - Implantation of Eu into SiO2

Before implantation into the fibre, we determined the optimal fluence range using a Silicon substrate with a 90-nm thermal SiO_2 film on top. The choice of substrate was motivated by the composition of the fibre core being similar to it. As per the previous experiments a thin film of 15 nm Europium was evaporated and irradiated with increasing fluences as follows, **1:** 1.6×10^{12} cm^{-2}, **2:** 2.4×10^{12} cm^{-2}, **3:** 3.6×10^{12} cm^{-2}, **4:** 5.4×10^{12} cm^{-2}, **5:** 8.1×10^{12} cm^{-2}, **6:** 1.2×10^{13} cm^{-2}, **7:** 1.8×10^{13} cm^{-2}, **8:** 2.7×10^{13} cm^{-2}, **9:** 4.1×10^{13} cm^{-2}, **10:** 6.2×10^{13} cm^{-2}, **11:** 9.2×10^{13} cm^{-2}, **12:** 1.4×10^{14} cm^{-2}, **13:** 2.1×10^{14} cm^{-2}, **14:** 3.1×10^{14} cm^{-2}, **15:** 4.7×10^{14} cm^{-2}. After irradiation, the Eu thin film was removed by dilute HCl at 50 °C for 30 min. The sample was then studied in PL without further post processing. A PL map of the regions irradiated is shown in Supplementary Figure 16a. Spectra of the irradiated regions are shown in Supplementary Figure 16b. Notably, the emission is unchanged throughout the fluence series with a prominent peak at ~620 nm, corresponding to the $^5D_0 \rightarrow {}^7F_2$ transition of the Eu^{3+} ion. The fluence yielding the highest PL intensity was then used for implantation into the optical fibre.

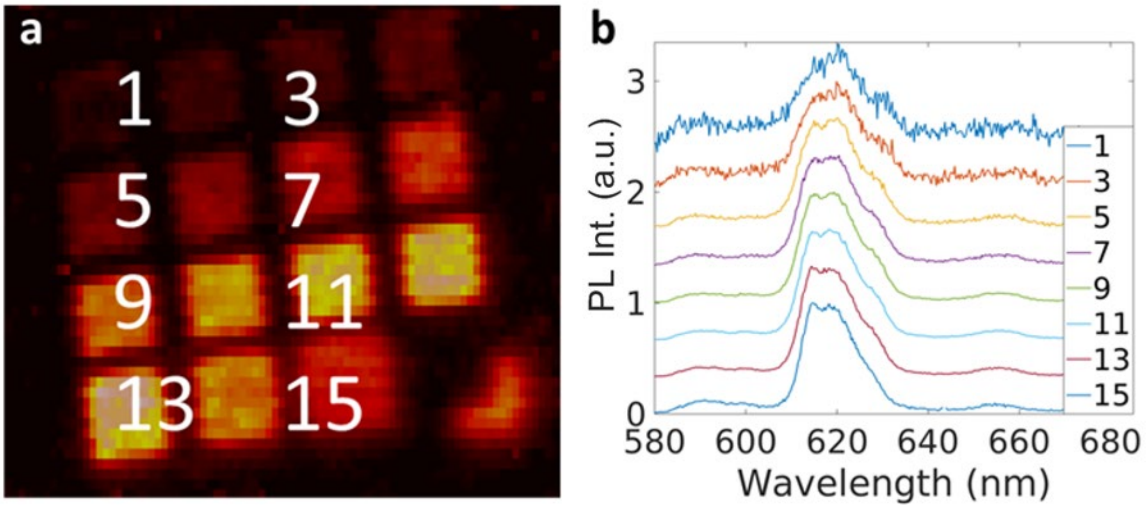

Supplementary Figure 16. *Implantation of Eu into SiO_2. a) Fluence series with values as mentioned in the main text. b) Normalised Spectra from the square regions.*

Supplementary References

1. Schröder, T.; Trusheim, M. E.; Walsh, M.; Li, L.; Zheng, J.; Schukraft, M.; Sipahigil, A.; Evans, R. E.; Sukachev, D. D.; Nguyen, C. T.; Pacheco, J. L.; Camacho, R. M.; Bielejec, E. S.; Lukin, M. D.; Englund, D., Scalable Focused Ion Beam Creation of Nearly Lifetime-Limited Single Quantum Emitters in Diamond Nanostructures. *Nat. Commun.* **2017,** *8*, 15376.
2. Zhou, Y.; Mu, Z.; Adamo, G.; Bauerdick, S.; Rudzinski, A.; Aharonovich, I.; Gao, W.-b., Direct Writing of Single Germanium Vacancy Center Arrays in Diamond. *New Journal of Physics* **2018,** *20*, 125004.
3. Rugar, A. E.; Lu, H.; Dory, C.; Sun, S.; McQuade, P. J.; Shen, Z.-X.; Melosh, N. A.; Vučković, J., Generation of Tin-Vacancy Centers in Diamond Via Shallow Ion Implantation and Subsequent Diamond Overgrowth. *Nano Lett.* **2020,** *20*, 1614-1619.
4. Iwasaki, T.; Miyamoto, Y.; Taniguchi, T.; Siyushev, P.; Metsch, M. H.; Jelezko, F.; Hatano, M., Tin-Vacancy Quantum Emitters in Diamond. *Phys. Rev. Lett.* **2017,** *119*, 253601.

5. Ziegler, J. F.; Ziegler, M. D.; Biersack, J. P., Srim – the Stopping and Range of Ions in Matter (2010). *Nuclear Instruments and Methods in Physics Research Section B: Beam Interactions with Materials and Atoms* **2010,** *268*, 1818-1823.
6. Ziegler, J. F.; Ziegler, M. D.; Biersack, J. P., 8 - Trim – Setup and Input *http://www.srim.org/SRIM/SRIM%2008.pdf* **2008**.
7. Bahm, A., Predictive Modelling of Gas Assisted Electron and Ion Beam Induced Etching and Deposition. *http://hdl.handle.net/10453/62155* **2016**.
